# Knocking on Cells’ Door: Strategic Approaches for miRNA and siRNA in Anticancer Therapy

**DOI:** 10.3390/ijms26178703

**Published:** 2025-09-06

**Authors:** Massimo Serra, Alessia Buccellini, Mayra Paolillo

**Affiliations:** Department of Drug Sciences, University of Pavia, 27100 Pavia, Italy; massimo.serra@unipv.it (M.S.); alessia.buccellini01@universitadipavia.it (A.B.)

**Keywords:** miRNA, siRNA, anticancer therapy, molecular design, targeted delivery

## Abstract

Metastasis is the main cause of failure in anticancer therapies, and is frequently related to poor prognosis for patients. The true challenge in extending cancer patient life expectancy, eventually managing cancer as a chronic disease with periodic but controllable relapses, relies on the development of effective therapeutic strategies specifically targeting key mechanisms involved in the metastatic cascade. Traditional chemotherapy with alkylating agents, microtubule inhibitors, and antimetabolites has shown limited efficacy against metastatic cells, largely due to the emergence of chemoresistant populations that undergo epithelial-to-mesenchymal transition (EMT), promoting the colonization of distant organs and sustaining metastatic progression. This scenario has spurred significant efforts to identify small molecules and biologics capable of interfering with specific steps in the metastatic process. In this review, we provide an overview of recent advances involving small interfering RNAs (siRNAs) and microRNAs (miRNAs) in cancer therapy. Although most of these agents are still under investigation and have not yet been approved for clinical use, insights into their development stage offer valuable information to identify new targets in the ongoing fight against metastasis. Particular emphasis is placed on the role of chemical modifications applied to siRNAs, such as backbone, sugar, terminal, base, and conjugation changes, and how these factors influence their stability, immunogenicity, and targeting precision. By integrating these aspects into the discussion, this review provides a focused and up-to-date resource for researchers in medicinal chemistry, drug delivery, and pharmaceutical formulation, where molecular design plays a critical role in therapeutic success.

## 1. Introduction

mRNA technology, together with innovative drug delivery solutions, has opened new pathways in pharmacological research; mRNA’s role in vaccine design, targeting either viral or tumor antigens, is continually gaining prominence. Alongside these trends, the possibility of modulating gene expression from within the cell, by regulating RNA translation and editing, has always attracted scientists from various fields, including clinical research. The finding that mRNA expression can be modulated by micro RNAs (miRNAs), with profound implications on post-transcriptional regulation and eventually gene expression, suggested the idea that this way of modulating gene and oncogene expression could be exploited as a therapeutic strategy. This strategy can be considered the opposite side of the therapeutic design aimed at blocking proteins, e.g., receptors or enzymes, by using chemical or biological agents: in this latter case, in fact, the gene product, after translation and post-translational modifications, is inhibited in its cellular functions by small molecules or antibodies. The discovery of miRNAs suggested that the target protein could be inhibited by selectively blocking its synthesis using synthetic, targeted RNAs named short interfering RNAs (siRNAs) or by directly targeting miRNAs, making other agents virtually unnecessary, with evident advantages under the side effect profile.

The functions of miRNAs, indeed, appear to be complex; miRNAs bind to target mRNAs, generating a double-strand RNA that cannot undergo the following translation process, eventually resulting in the degradation or inhibition of the target mRNA. The functions of miRNAs are complex, since a single miRNA can bind to and regulate a wide range of mRNAs, and entire cellular pathways can be modulated by a specific miRNA. This modulation can lead to relevant biological outcomes and deeply influence the cell’s fate. For this reason, miRNAs immediately appeared to be a potential pharmacological tool, and the importance of these fine intracellular regulators was recognized by the awarding of the 2024 Nobel Prize in Physiology or Medicine to Victor Ambros and Gary Ruvkun, who first identified and described them.

## 2. Micro RNAs (miRNA)

miRNAs are typically located in the introns or exons of non-coding RNA sequences, or can be found in the introns of pre-mRNAs [1]. miRNAs are generally transcribed by RNA polymerase II (Pol II) as primary transcripts, called pri-miRNAs, which are long sequences containing stem-loop structures composed of approximately 70 nucleotides each [2]. Once in the cell nucleus, pri-miRNAs are cleaved by the Drosha/DGCR8 complex into precursor miRNAs (pre-miRNAs), which retain their characteristic hairpin structure [2]. The pre-miRNAs are then translocated into the cytoplasm by a family of proteins known as Exportins, and subsequently bound and cleaved, near the loop, by ribonuclease III Dicer to form a mature miRNA duplex of about 22 nucleotides in length [2]. This latter small double-stranded RNA (dsRNA) is finally loaded into the Argonaute 2 (Ago2) protein and then the guide strand is selected and charged into the RNA-induced silencing complex (RISC) along with the Ago protein [3]. While the passenger strand is unwound, released, and degraded by the Ago, the seed region of the guide strand, comprising the first nucleotides from its 5′ end, binds to the complementary 3′ untranslated region (3′ UTR) of target mRNAs [3], leading to the regulation of gene expression through mRNA cleavage, degradation, and/or translational repression [4]. However, either strand can be loaded into RISC; arm switching may occur due to regulatory factors that remodel the miRNA duplex or influence Ago protein strand-loading preferences in a context-dependent manner. Such factors may induce conformational changes in Argonaute or, as in the case of DNA damage observed in cancer, alter strand preference and selection. Nevertheless, Ago2 usually prefers the strand with a 5′ uracil that originates from the less stable end of the duplex, which typically corresponds to the antisense strand [5].

In fact, this regulatory mechanism is crucial for maintaining normal cellular functions, and the dysregulation of miRNAs is implicated in a wide range of diseases, including cancers. Some miRNAs, called oncomiRs, are overexpressed in tumors and promote cancer progression and metastasis by repressing tumor-suppressor genes. For example, the miR-17-92 cluster and miR-21 are frequently upregulated in various cancers such as lung, colon, breast, and ovarian cancers, where they target mRNAs coding key tumor suppressors like PTEN and PDCD4, enhancing tumor growth and drug resistance [6,7]. Conversely, some others function as tumor-suppressor miRNAs (TS-miRNAs), and their downregulation can lift inhibitory constraints on tumor progression. Members of the let-7 family, miR-29, and miR-34 are well-characterized TS-miRNAs, and their loss correlates with poor prognosis in cancers, including lung, breast, and pancreatic tumors [6,7].

Therefore, an innovative therapeutic approach involves the combined administration of anti-miRNAs, designed to specifically bind and neutralize aberrantly overexpressed oncomiRs, and miRNA mimics, aimed at restoring the activity of tumor-suppressor miRNAs [8]. Indeed, in a murine model of hepatocellular carcinoma (HCC), the combination of miR-122 mimic and anti-miR-221 reduced tumor growth much more effectively than the administration of either molecule alone [9]. This represents a promising alternative to traditional therapies for HCC and, potentially, for other solid tumors.

## 3. Short Interfering RNAs (siRNAs)

**Figure 1:**
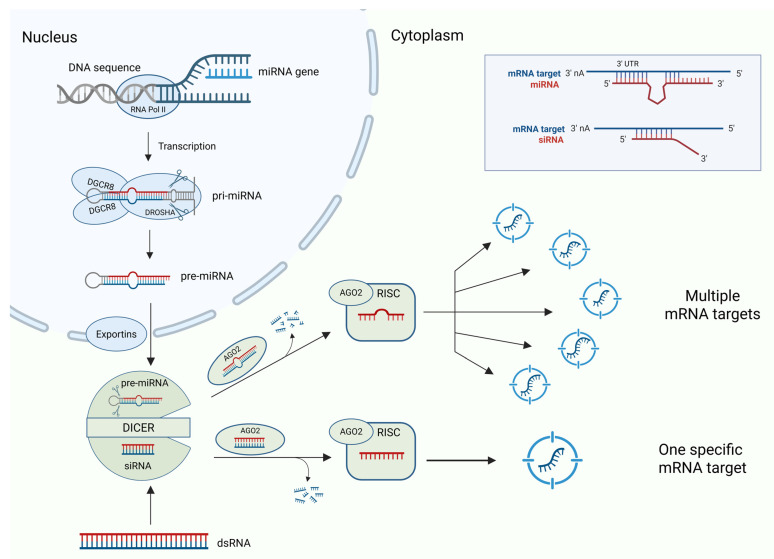
miRNA and siRNA: biogenesis and targets. Endogenous miRNAs target and regulate multiple mRNA expressions due to their loop structures, whereas synthetic siRNAs specifically target and regulate single mRNA expression.

siRNAs, also known as short interfering RNAs, are a type of non-coding double-stranded RNAs of 20–23 nucleotide base pairs in length that act by interfering with the expression of the specific gene having short stretches complementary to sequences to target mRNA [10]. siRNAs are similar to endogenous microRNAs (miRNAs) in terms of functions, except that microRNAs can regulate the expression of hundreds of genes via imperfect base-pairing whereas siRNAs bind more specifically to the single mRNA at a particular location. The significant and peculiar difference between these two non-coding RNAs is that the miRNA has multiple targets, whereas siRNA has only one specific mRNA target (Figure 1).

The great advantage of siRNA as a drug therapy platform, compared with other conventional drugs, such as small molecules or antibodies, relies on their capability to be taken up by target cells, processed by the RNA-induced silencing complex (RISC) to release single-strand RNA (ssRNA) that, in turn, degrades its complementary mRNA, thereby blocking the translation of the target protein [11,12]. The intrinsic structure of non-modified naked siRNA therapeutics, together with the hurdle to overcome systemic delivery issues, poses some critical and important challenges regarding their bioavailability (such as serum stability), off-target effects, and ability to trigger an immunogenic unwanted response [13]. Efforts to cope with these challenges identified two founding pillars required for a successful siRNA drug therapy. The first pillar relies on the modification of the structure of siRNAs to limit their degradation, increasing their stability and at the same time improving their binding affinity toward target mRNAs [14].

The second pillar relies on the design of delivery systems to enhance the amount of siRNA that enters the target cell [15]. Indeed, naked siRNAs are polar and poor lipophilic compounds that do not cross cell membranes, thus requiring a lipophilic “shuttle” system to enhance their entrance and subsequent bioavailability inside cells.

### 3.1. Beyond Native siRNA: A Chemical Optimization Guide

**Figure 2:**
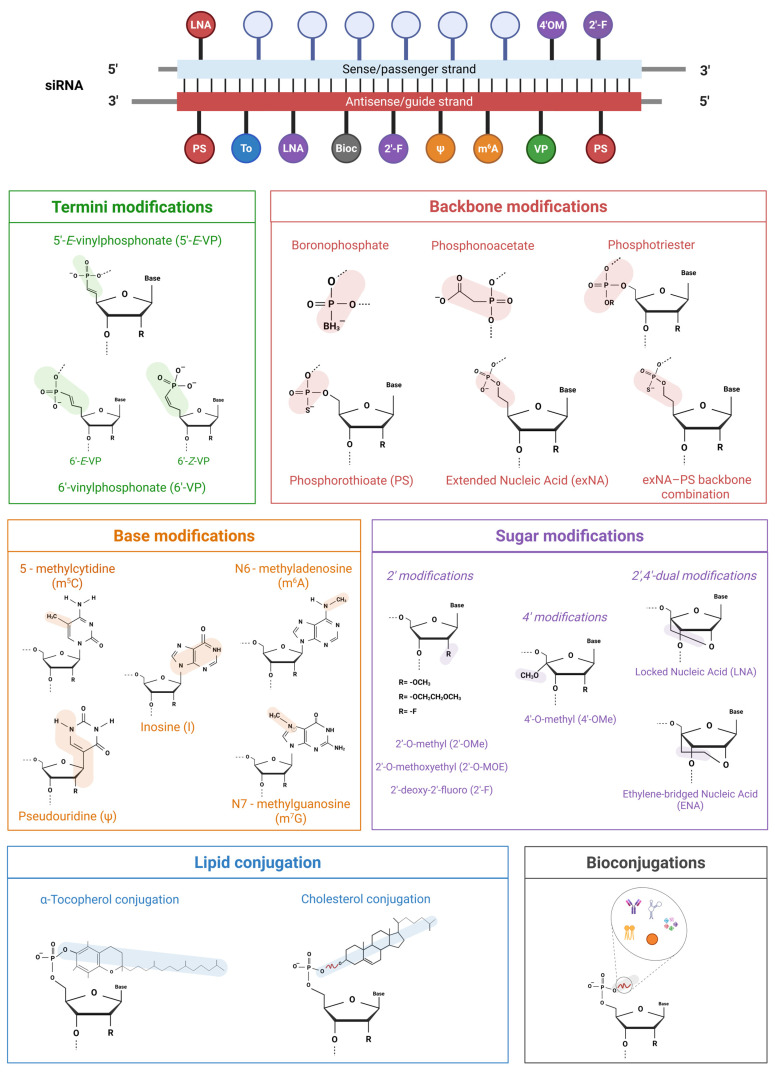
Schematic representation of the most common types of siRNA modifications. siRNAs are typically optimized at the antisense (guide) strand to enhance RNA interference. However, certain chemical modifications can also be applied to the sense (passenger) strand to prevent its incorporation into the RISC machinery, improve stability, or reduce off-target effects.

In recent years, siRNAs have undergone a broad range of structural modifications aimed at enhancing their biopharmaceutical properties (Figure 2). Several studies have focused on the simultaneous substitution of chemically modified monomers or functional groups to evaluate their effects on siRNA activity and cytotoxicity. The main objective of these modifications is to overcome the limitations of unmodified siRNAs without compromising their activity [16] and, ultimately, to ensure that they achieve optimal performance in vivo, where stability, distribution, immunogenicity and on-target efficacy are critical [17].

#### 3.1.1. Termini Modifications

The addition of a phosphate group at the 5′ end of the siRNA guide strand is essential for recognition by Ago2 and processing by RISC machinery that mediates siRNA unwinding and target mRNA pairing. In fact, the 5′ phosphate is arguably the most critical structural element for strand selection and Ago2 loading. Once in the cell, siRNAs are dynamically regulated by Clp1 kinase and phosphatases, which maintain a balance between the 5′ phosphorylated and dephosphorylated (hydroxyl-terminated) ends [18]. Modifications at the 5′ terminus can influence the activity of these enzymes by altering the phosphorylation status and, consequently, the likelihood of a guide strand being incorporated into the RISC complex. On the other hand, such modifications may also lead to recognition by phosphorylases, potentially resulting in decreased stability and reduced gene silencing efficacy [18]. So, caution is warranted when using extensively modified siRNAs. To overcome these limitations, non-hydrolysable 5′ phosphate analogues have been developed, most notably the 5′-(*E*)-vinyl-phosphonate group (VP) [19]. These analogues mimic the natural phosphate moiety while resisting enzymatic hydrolysis, allowing it to stably occupy the deep 5′ phosphate-binding pocket of Ago2 [18,19] and improving metabolic stability. According to a 2016 study by Jadhav [20], siRNA modified with 5′-E-VP exhibited up to a 20-fold increase in potency in vitro and a 3-fold increase in RNA interference (RNAi) activity in vivo in a mouse model targeting apolipoprotein B (ApoB). Moreover, a recent update [21] showed that modification at the 6′ position of the vinyl-phosphonate (6′-VP) on the siRNA antisense strand can further enhance RNAi activity. Unlike 5′-VP, where only the E isomer is effective, both E and Z isomers of 6′-VP increase siRNA potency. This modification bypasses the strict stereochemical constraints of the 5′ position, enabling the broader application of VP isomers in siRNA design.

#### 3.1.2. Backbone Modifications

The stability and in vivo bioavailability of siRNAs can be improved by replacing the standard phosphodiester linkage with boron, sulfur, or acetate, thus forming boranophosphate, phosphorothioate (PS), and phosphonoacetate linkages, respectively. The presence of these exogenous linkages enhances the resistance of siRNAs to both 5′ and 3′ exonuclease hydrolysis and increases their binding affinity to plasma proteins, resulting in a prolonged siRNA half-life in circulation [12], reduced renal clearance, and increased hepatic accumulation [22]. These properties are particularly advantageous for liver-targeted delivery. The liver displays unique features that make the accumulation and internalization of siRNAs easier. For instance, it is one of the most vascularized organs and highly expresses specific receptors, such as the asialoglycoprotein receptor (ASGP-R), which can be targeted by triantennary N-acetylgalactosamine (GalNAc)-conjugated siRNAs [23]. Otherwise, more strategies are required to overcome hepatic entrapment and enable effective accumulation in extrahepatic tissues, such as solid tumors [24]. In this regard, a new modification called extended nucleic acid (exNA) has recently been developed in combination with the PS backbone, involving the insertion of a methylene group between the 5′ carbon and the 5′ hydroxyl group of a nucleoside, and subsequently applied to siRNA. The synergic action of the exNA–PS backbone combination has been able to significantly increase resistance to exonucleases, up to 32 times more than the PS backbone alone, thus enhancing stability, accumulation, and, in turn, siRNA efficacy in vivo [25]. Another strategy involves the temporary replacement of phosphodiester backbone with phosphotriester group. This modification enhances the cellular uptake of siRNAs by neutralizing their negative charge, generating small interfering ribonucleic neutrals (siRNNs) that are then converted into native charged phosphodiester-backbone siRNAs by the action of cytoplasmic esterases. It also increases their half-life in circulation and strengthens RNA interference [26]. An example is the siRNN targeting Polo-like kinase 1 (Plk1), which is highly overexpressed in pediatric patients with B-cell acute lymphoblastic leukemia (B-ALL). The target specificity of the siRNA, developed as an RNAi prodrug, enabled the effective suppression of Plk1 mRNA and protein levels while minimizing off-target effects [27].

#### 3.1.3. Sugar Modifications

The 2′-hydroxy group of the ribose sugar can be replaced by less nucleophilic moieties to gain 2′-O-methyl (O-Me), 2′-O-methoxyethyl (MOE) and 2′-fluoro (F) derivatives. The main objective of these modifications is to prevent 2′-OH-mediated hydrolysis, thereby increasing resistance to endonucleases while preserving the C3′-endo ribose conformation, which is essential for RISC recognition and thermal stability of siRNA duplex [28]. Indeed, a major advantage of sugar modifications compared with backbone modifications is their inability to affect siRNA recognition by RISC. In addition, replacing the 2′-OH group is essential for preventing innate immune activation [29,30]. The effect of the 2′-modification can be further improved by adding a 4′-modification. The most potent sugar modification thus involves a combination of 2′ and 4′substitutions, placed at internal or terminal positions of the guide strand or the duplex, yielding a 2′,4′-dual modification [31]. Currently, several combinations have been tested in vitro on cancer cells to evaluate their RNAi activity, for an eventual translation in in vivo anticancer therapy. For instance, siRNA targeting the downregulated in renal cell carcinoma (DRR) gene, modified with 2′-F and 4′-OMe at the overhangs, exhibited excellent activity (IC_50_ ~ 4 nM) under the tested conditions [31]. 2′-OMe-4′-thioRNA targeting the proto-oncogene serine/threonine protein kinase (RAF-1) was 5-fold more potent than unmodified siRNA after 6 days of silencing (IC_50_ 118 nM) [32]. Moreover, the sugar ring can also be extensively modified. Bridged nucleic acids (BNAs), in which a methylene or ethylene bridge links the 2′-oxygen and the 4′-carbon of the ribose, constrain the sugar conformation and give rise to locked nucleic acids (LNAs) and ethylene-bridged nucleic acids (ENAs), respectively. The use of locked sugar modifications in siRNAs leads to increased thermal and nuclease stability, resulting in enhanced binding affinity for the target mRNA. However, these modifications are highly position-sensitive. While many LNA insertions are well-tolerated, modifications near the RNA cleavage site markedly reduce silencing activity, likely due to conformational or functional effects on the RISC complex [33]. In contrast, LNA at the 5′ end of the sense strand promotes preferential antisense strand loading, thereby reducing sequence-related off-target effects [33,34].

#### 3.1.4. Base Modifications

Immune cells can distinguish foreign synthetic siRNAs from endogenous ones because synthetic siRNAs often lack naturally occurring chemical modifications, such as 2′-O-methylation or base methylation, that help endogenous RNAs to evade innate immune sensors in mammalian cells. This recognition is predominantly mediated through the activation of toll-like receptor (TLR) pathways and can lead to undesirable side effects due to the significant toxicities associated with excessive pro-inflammatory cytokines release [35]. The immune response is triggered by the presence of the 2′-OH group on the ribose, and by the siRNA sequence itself. Therefore, base substitutions with modified nucleotides are a potential tool to mask exogenous siRNAs from the immune system [29]. Natural nucleobase analogues include, for instance, pseudouridine, 5-methylcytidine, N6-methyladenosine, inosine, and N7-methylguanosine. Modifications near the 5′ end of the guide strand seem particularly useful, especially in sequences rich in adenosine, which is known to be immunostimulatory [29]. However, there are currently not many significant examples of base substitution in the design of siRNAs compared with other, more common modifications. N6-methyladenosine (m^6^A) modification has been tested in combination with sugar modifications to be effective in silencing F7 gene (coagulation factor VII) in HuH7 human hepatocarcinoma cells [36]. Moreover, the use of base modifications raises safety concerns, particularly for unnatural nucleobase analogues such as N-ethylpiperidine 7-ethynyl-8-aza-7-deazaadenosine triazole (NEP7EAA). These compounds are incorporated into synthetic oligonucleotides and must be safely metabolized by the human body [37]. Another class of base modification involves abasic mimics, which lack nucleobases entirely. This strategy replaces a nucleotide in the guide strand, specifically at the pivot position (AS6), with an abasic spacer. By preventing base pairing at this critical site, abasic substitutions effectively eliminate miRNA-like off-target effects in vivo, while preserving robust on-target gene silencing [38]. Unlike other base modifications, abasic substitutions do not trigger TLR-mediated innate immune responses [38].

#### 3.1.5. Lipid Conjugation

The conjugation of lipids to the 5′ or 3′ termini of siRNA enhances their binding to serum proteins and promotes cellular uptake, thereby enabling carrier-free delivery and improving tissue distribution, and overall in vivo bioavailability. Specifically, conjugation with lipids, such as cholesterol, diacyl lipid and vitamin E, significantly increases siRNA uptake in several tissues upon intravenous injection. The high affinity for serum proteins, such as albumin or lipoproteins (LDL/HDL), prolongs siRNA circulation time and slows its distribution. This facilitates tissue-specific internalization via recognition by cell surface receptors (e.g., LDLR, SR-B1, especially for targeting the liver), membrane fusion, or endocytosis, and protects siRNA from renal filtration, thereby leading to more tumor penetration [39]. Several siRNAs are cholesterol-conjugated (Ch-siRNAs), one example of them for anticancer therapy is the anti-MDR1 siRNA (Ch-siMDR), designed by binding cholesterol via a linker and incorporating a 2′-O-methyl modification to prevent enzymatic degradation. This siRNA silences P-glycoprotein expression and restores vinblastine sensitivity in breast cancer models in vitro and in vivo [40]. Furthermore, a diacyl lipid modified siRNA (siRNA-L2) was tested for triple negative breast cancer in vitro and in vivo. By leveraging albumin as an endogenous carrier, the bioavailability of siRNA is significantly increased compared with that of unmodified siRNA, and the tumor-to-liver accumulation ratio is markedly more favorable compared with siRNA delivered with traditional nanoparticles [39]. Lipid conjugation is particularly advantageous for liver targeting. The strong lipophilic nature of vitamin E has been exploited to design α-tocopherol-bound modified siRNAs (Toc-siRNAs), harnessing its physiological pathways, such as incorporation into lipoprotein, for the targeted delivery to apolipoprotein B (apoB) in liver cancer [41].

#### 3.1.6. Bioconjugations

Bioconjugation has recently emerged as one of the most innovative and promising approaches to bypass hurdles and problems associated with direct chemical modifications of siRNAs [42]. The principle of this approach relies on the formation of a covalent binding between siRNA and a variety of other biogenic molecules, such as antibodies, aptamers, carbohydrates, lipophilic derivatives, oligonucleotides, peptides, and polymers. Bioconjugates display several advantages over classical non-conjugated nanosystems such as lipid nanoparticles, polymeric nanoparticles, metallic core nanoparticles, dendrimers, and polymeric micelles. Indeed, siRNA-bioconjugate complexes could be assembled without a positive charge, avoiding the need for cationic carriers to neutralize the siRNA’s negative backbone. They are less immunogenic due to their small size, and furthermore, the chemical nature of the linkage (e.g., cleavable or photosensitive bonds) between siRNA and its cognate bioconjugate can be tailored to modulate stability, efficiency, accumulation, and resistance to RNase. Examples of siRNA bioconjugates with peptide or protein ligands include those designed to target receptors that are highly overexpressed in cancer, such as CD47, PD-L1, and EGFR. These ligands guide the siRNA to cancer cells through receptor-mediated recognition and internalization. Once inside the cytoplasm, the siRNA silences the mRNA encoding the same receptor, thereby reducing its expression and enhancing therapeutic efficacy. Successful in vitro results were obtained in various cancer cell lines [43], with the aim of translating these findings into in vivo models. However, the main limitation of siRNA bioconjugate is related to the lack of specificity of the various conjugated chemical moieties, which can hamper the recognition of specific targets on the membranes of recipient cells. Although this drawback may not be critical when high delivery selectivity is not required and accumulation in non-target cells does not cause side effects, future studies are essential to understand the interaction between conjugates and target surface molecules. Such studies will be crucial to boost the clinical application of siRNA bioconjugates.

### 3.2. siRNA Delivery

**Figure 3:**
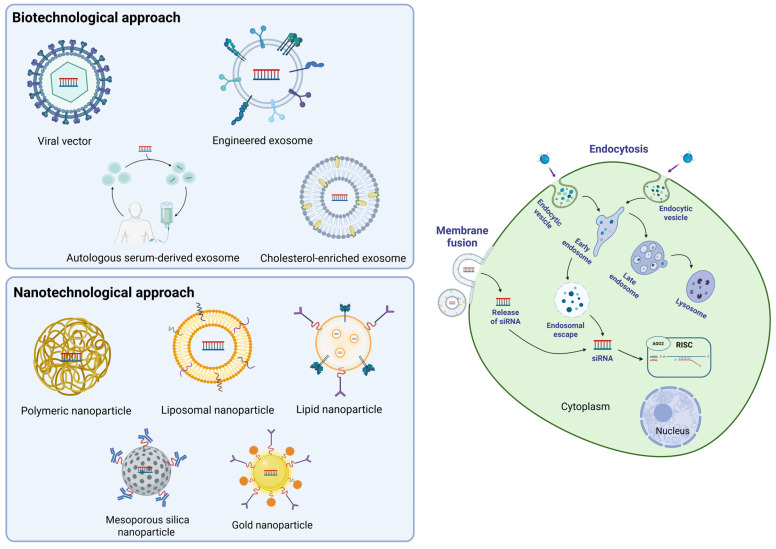
Illustration of delivery systems based on biotechnological and nanotechnological approaches and their main cellular entry mechanisms. These platforms act as “shuttle” systems to enhance uptake and intracellular release via endocytosis or membrane fusion. The main objective is to achieve siRNA internalization and silencing of its target mRNA.

siRNA delivery systems can be roughly divided into two big families: i. delivery systems developed using a biotechnological approach, which involve the use of both viral vectors and non-viral platforms, such as small extracellular vesicles (EVs); and ii. delivery systems developed using a nanotechnological approach, which include nanoparticles, liposomes, and other nanosystems (Figure 3).

#### 3.2.1. Biotechnological Approach

Viral vectors display optimal properties as siRNA delivery systems: they combine high transfection efficiency with the ability to specifically target sensitive cells that may not tolerate the harsh conditions associated with other delivery methods (e.g., cationic lipids, electroporation) [15]. However, the implementation of siRNA incapsulated in viral vectors is a rather expensive and complex procedure that necessitates trained staff with good skills in molecular biology and virology. In addition, safety concerns in terms of the ability to induce dangerous mutations or unwanted incorporation of viral genes into the host DNA [44], together with the risk of triggering immunogenic and inflammatory processes, have slowed attempts to set up the production of safe vectors for human use. These limitations have therefore shifted efforts in this field toward the development of non-viral delivery systems, mainly extracellular vesicle (EV)-based systems.

Extracellular vesicles (EVs) are cell-secreted particles that differ in size, shape, content, biogenesis, and structure, and are principally subclassified into exosomes (EXs), microvesicles, and apoptotic bodies, ordered from the smallest to the largest [45]. EVs are currently one of the most investigated shuttle systems due to their potential to be easily loaded with small-molecule cargos combined with their supposed ability to be selectively and specifically taken up by different cell types. Notably, the selective uptake of EVs by target cells could be further fine-tuned by functional modification of the molecules expressed on the EVs membrane that are supposed to interact with their cognate counterparts present on the membranes of target cells. A notable example of chemical surface modification of exosomes involves a membrane engineering strategy using donor cells [46]. In this approach, donor cells are exposed to DSPE-PEG-RGD, a phospholipid–RGD conjugate that spontaneously embeds in the plasma membrane and is subsequently incorporated into the exosome membrane. The resulting RGD-functionalized exosomes exhibit enhanced cellular uptake via receptor-mediated endocytosis through αvβ3 integrin, which is highly expressed on angiogenic endothelial and tumor cells. Another strategy involves using HEK293F cells genetically engineered to overexpress the integrin subunits ITGAL and ITGB2, which combine to form the LFA-1 receptor on the surface of released EVs. This surface modification enables selective binding and internalization by endothelial cells that highly express ICAM-1, an adhesion molecule upregulated during inflammation [47]. Alternatively, a recent cell donor-free strategy relying on enzymatic glycoengineering enables the chemical functionalization of exosomes by incorporating azido-sialic acids into their surface glycans. These azido groups serve as versatile chemical handles for ligand attachment via click chemistry, allowing efficient and customizable targeting of specific cells [48].

Generally, engineered exosomes offer several advantages for enhancing the cytosolic delivery of siRNA molecules, which must interact with the RISC complex to achieve effective gene silencing. Exosomes derived from human liver cancer cells (HepG2) are naturally enriched in membrane cholesterol. This feature has recently been studied for its ability to improve exosome uptake by cancer cells via membrane fusion rather than endocytosis, thereby bypassing endolysosomal entrapment and optimizing transportation efficiency. Building on this natural property, cholesterol-enriched milk-derived exosomes (e.g., 30%Chol-MEs) loaded with siPLK1 have effectively suppressed tumor growth in subcutaneous HCT116 colorectal cancer-bearing mice, without inducing organ toxicity and eliciting a strong immune response [49].

The discovery that cancer-derived exosomes preferentially target distant organs prompted researchers to pursue this strategy in animal models of cancers with high metastatic potential. Autologous serum-derived exosomes, obtained from mice injected with melanoma cells, loaded with siRNA directed against the heparan sulfate proteoglycans Glypica-3 (GPC3), significantly reduced the spread of tumor cells as assessed by the number of metastatic lung colonies [50].

Surface engineering of autologous exosomes represents a promising strategy to improve their selective targeting capacity for siRNA delivery. Among recent approaches, the development of CBSA/siS100A4-exosomes has shown notable potential. CBSA/siS100A4-exosomes are membrane-coated lipid nanoparticles (LNPs) incorporating cationic bovine serum albumin (CBSA) complexed with siRNA targeting S100A4. This biomimetic system enhances siRNA protection from degradation and improves biocompatibility compared with conventional conjugated LNPs, leading to more efficient silencing of the metastasis-associated protein S100A4 and reduced proliferation of triple-negative breast cancer (TNBC) cells [51].

Another example of biomimetic engineering involves apoA1-bExo, an exosome functionalized with the tumor-targeting apolipoprotein A1 (apoA1), used to deliver siRNA targeting neutral sphingomyelinase 2 (nSMase2), an enzyme implicated in exosomal PD-L1 secretion. Inhibiting this pathway is critical for restoring T cell function and enhancing the efficacy of immune checkpoint blockade therapies, potentially limiting tumor progression and resistance. In a HepG2 tumor-bearing humanized mouse model, apoA1-bExo enabled improved tumor selectivity and cellular uptake via the scavenger receptor class B type 1 (SR-B1), facilitating efficient cytosolic delivery of siRNA through an apoA1-mediated selective uptake mechanism [52].

Exosome-based siRNA delivery has also been employed to silence non-canonical long non-coding RNA (lncRNA) involved in the progression of TNBC [53]. Specifically, the overexpression of lncRNA DARS-AS1 modulates the migration and invasion of TNBC tumors by inhibiting miR-129-2-3p via upregulation of the CDK1 kinase. Treatment with exosomes loaded with siRNA targeting DARS-AS1 substantially reduced TNBC cell growth and liver metastasis, thus confirming that siRNA-loaded exosomes represent a promising tool in contrasting highly metastatic tumors.

#### 3.2.2. Nanotechnological Approach

Polymeric nanoparticles, metallic core nanoparticles, dendrimers, and polymeric micelles have been extensively exploited in recent years and are currently under investigation as promising delivery systems. Indeed, these non-liposomal siRNA nanocarriers are quite stable, and their surface can be easily chemically modified to increase their transport and target efficiency. A wide array of biocompatible and non-toxic materials, including natural polymers such as chitosan and cyclodextrin, as well as synthetic polymers like polyethyleneimine (PEI) and polylactic-co-glycolic acid (PLGA), have been successfully tested and proven to be safe and efficient in encapsulating nanoparticles for siRNA delivery [54,55], while also offering advantages in terms of pharmacokinetics. In a recent study, a combination of poly (ethylene glycol)-b-poly (d,l-lactide) (mPEG-b-PLGA) and cationic lipids (iCLAN) was used to encapsulate siRNA achieving over 95% siRNA loading efficiency. iCLAN nanoparticles carrying siCD47 triggered a strong immune response that suppressed melanoma tumor growth by blocking a critical immune checkpoint, thereby highlighting the use of nanoparticles in cancer immunotherapy [56]. In another appealing perspective, chitosan-based nanoparticles have demonstrated good mucoadhesive properties and the ability to cross biological barriers, such as the blood–brain barrier (BBB), making them appealing candidates for targeting delivery to brain tumors or for the treatment of neurodegenerative diseases [55].

Unfortunately, the downside of nanocarriers lies in the complexity of their design, which requires the careful selection and combination of suitable materials, as well as the necessary chemical modifications to obtain a reliable and efficient delivery system.

Lipid nanoparticles (LNPs) are widely used and clinically validated systems for siRNA delivery. Their lipid envelope protects siRNA from nuclease cleavage, thus enabling the therapeutic cargo to reach specific organs or cellular targets.

Depending on their charge properties, siRNA-binding lipids could also be classified into three main classes: cationic, neutral, and ionizable LNPs [57,58].

Both ionizable and cationic LNPs can interact with circulating lipids and plasma proteins, which often results in preferential accumulation in hepatocytes or retention in the endosome and lysosome compartments. A remarkable example is Patisiran: this is a clinically approved RNA interference (RNAi) therapeutic that uses (LNPs) containing the ionizable lipid DLin-MC3-DMA to deliver siRNA targeting transthyretin (TTR) for the treatment of hereditary ATTR amyloidosis [59]. Those lipids exhibit a positive charge under acid conditions, and a neutral charge under physiological pH conditions. This characteristic confers protection against degradation, enhances cellular uptake, and enables specific downregulation of gene expression [59]. An interesting aspect is the ability of LNPs to cross the BBB: in addition to delivering specific siRNAs, they offer a significant advantage due to their high loading capacity, which enables therapeutic effects, even with a minimal fraction of siRNA reaching the brain. Preclinical studies have demonstrated the effectiveness of LNPs in delivering siRNA or mRNA to neuronal cells, reducing the expression of NMDA receptors [60] or showing therapeutic potential for neurodegenerative diseases such as Friedreich’s ataxia [61]. Furthermore, an interesting perspective is that the passage of cationic LNPs carrying specific siRNAs across the BBB could be achieved through intranasal administration, thereby avoiding systemic or intrathecal delivery. Indeed, cationic LNPs show some drawbacks that could make them less favorable for therapeutic applications, because of their higher toxicity and lower potency, mainly attributable to their higher nonspecific binding with plasma proteins and their ability to trigger immunogenic reactions. These unwanted properties have spurred efforts, both in the private sector and within public research institutions, to synthesize novel ionizable LNPs with improved safety profiles and optimized biodistribution, specifically designed to avoid hepatic accumulation. These considerations are particularly relevant when siRNA-LNPs are employed as a shuttle for antitumoral agents, where stringent selectivity is required to effectively target cancer cells while sparing healthy tissues.

Attempts to overcome these limitations involved the modification of LNPs’ surfaces with chemical moieties that could target molecules expressed on tumor cell outer membranes, in the tumor extracellular matrix (ECM), or in other non-tumor cell types located in the tumor microenvironment (TME), such as endothelial cells or tumor-associated macrophages (TAMs).

The most employed and screened tumor-targeting moieties that facilitate siRNA-loaded LNP accumulation in cancer cells include aptamers or other small molecules (hyaluronan or folate), short peptides (RGD-based peptides or octreotide), and large proteins or antibodies (transferrin) [62].

Liposomal nanoparticle (NP) platforms have been extensively investigated for siRNA delivery in cancer therapy, given their ability to suppress tumor cell proliferation and metastatic dissemination. Notably, PEGylated liposomal NPs incorporating carrier DNA and polycationic peptides enabled the co-delivery of siRNAs targeting MDM2, c-Myc, and VEGF in a murine lung cancer model, resulting in a marked inhibition of metastasis with minimal systemic immunotoxicity or off-target effects [63].

Additional strategies have focused on the use of liposomes loaded with siRNAs and surface-functionalized to target multiple signal transduction pathways involved in cancer cell metastasis. In metastatic breast cancer (MBC), C-X-C chemokine receptor type 4 (CXCR4) and lipocalin-2 (Lcn2) are critical mediators of cell migration. Liposomes con-jugated with anti-CXCR4 antibodies and loaded with siRNA against Lcn2 have demonstrated a synergistic inhibitory effect on cell migration in triple-negative human breast cancer cells [64].

A similar strategy exploits the chemical modifications of the surface of LNP with short peptides or chemical residues that are recognized by target cells, such as peptides incorporating the RGD sequence that binds several members of the integrin family overexpressed in a wide variety of cancer cells. An anti-angiogenic siRNA encapsulated in an RGD peptide-modified lipid nanoparticle (RGD-LNP) elicited a significant reduction in vascular permeability in a lung-metastasized animal model [65]. Notably, the cumulative anti-angiogenic effect induced by RGD-LNP in the metastasized lung was higher when compared with the analogous PEG-LNP, thus strongly indicating that this approach could be further refined to enhance the target specificity and cell permeability of surface modified LNP in the treatment of metastatic cancers.

The advantages of surface-modified lipid nanoparticles (LNPs) over non-modified formulations have been further demonstrated in a lung metastasis model [66]. In this study, self-assembling LNPs composed of an amphiphilic low-molecular-weight heparinursolic acid conjugate were encapsulated in PEG-lipids functionalized with anisamide, a ligand targeting the sigma receptor, which is overexpressed in metastatic lung tissue. siRNA-loaded, anisamide-targeted LNPs exhibited significantly enhanced gene silencing activity compared with both non-PEGylated and PEGylated untargeted controls. Moreover, confocal microscopy revealed that, unlike untargeted formulations, targeted LNPs preferentially accumulated in lung metastases with minimal off-target distribution.

The conjugation with an antibody recognizing an antigen on the surface of the target cell is another strategy for selective uptake into cancer cells. Antibody-conjugated mesoporous silica nanoparticles (MSNPs) coated with cross-linked PEI/PEG polymers were loaded with polo-like kinase 1 siRNA (siPLK1) and tested in an animal model of highly metastatic triple-negative breast cancer (TNBC) [67]. The authors reported that MSNPs inhibit cancer cell invasion via ROS- and NOX4-dependent mechanisms, reduce by 80% the level of PLK1 mRNA in metastatic breast cancer cells of mouse lungs, and increase the overall survival of treated animals.

The encapsulation of siRNA into synthetic nanoparticles (SNs) composed of PEG–polylactide copolymers has emerged as a promising strategy to enhance the efficacy of cancer therapies in combination with conventional anticancer agents. In a series of well-designed in vitro and in vivo experiments using a human pancreatic cancer model, the coadministration of siRNA-loaded SNs with arsenic-encapsulated vesicles resulted in a synergistic effect. Specifically, mutant KRAS silencing by siRNA combined with the pro-apoptotic and antiproliferative activity of arsenic significantly improved therapeutic outcomes [68].

Liver metastasis represents the most critical clinical challenge in patients with colorectal cancer (CRC), prompting recent efforts to develop siRNA-based formulations aimed at attenuating metastatic progression [69]. One of the major hurdles in oral siRNA administration is protecting the payload from gastrointestinal degradation while enabling active transport to achieve sufficient bioavailability. In this context, orally delivered gold nano-particle-based siRNA carriers, specifically AuNP-siRNA-glycol chitosan-taurocholic acid nanoparticles (AR-GT NPs) loaded with siRNA targeting Akt2, were shown to reduce endogenous Akt expression and induce apoptosis in a colorectal liver metastasis (CLM) animal model [70]. Other synthetic siRNA delivery systems based on polysaccharides and their derivatives have shown promising results in highly metastatic cancers, such as osteosarcoma. Notably, siRNA targeting astrocyte elevated gene-1 (AEG-1), encapsulated within the polysaccharide Amy-g-PLLD, was found to reduce osteosarcoma cell proliferation and invasion both in vitro and in vivo [71]. Furthermore, the direct injection of Amy-g-PLLD/siAEG-1 complexes into the tumor mass significantly suppressed lung metastases in tumor-bearing mice, without inducing detectable cytotoxic effects.

Beyond the classical approach of delivering chemically synthesized siRNA encapsulated in nanoparticles (NPs), an elegant alternative strategy seeks to overcome NP-associated toxicity by employing plasmid or viral vectors encoding short hairpin RNA (shRNA) precursors. These stem-loop RNA sequences, typically encoded by DNA vectors and introduced into cells via plasmid transfection or viral transduction, are subsequently processed by endogenous RNase enzymes, which cleave the loop to generate functional siRNA molecules. The Yes-associated protein (YAP) is overexpressed or constitutively activated in a wide variety of cancer types, where it regulates several mechanisms involved in metastatic processes. The silencing of YAP by shRNA induced tumor cell apoptosis and blocked tumor cell proliferation and angiogenesis in an animal model of gastric carcinoma [72]. Quite interestingly, YAP shRNA also decreases the expression of several other genes that support metastatic spreading, thus highlighting the utility of the shRNA approach in enhancing the efficacy and safety of siRNA delivery systems.

The debate over the superiority of one siRNA delivery system over others, in terms of efficiency and toxicity, is still an open question seldom addressed in the siRNA literature. The comprehensive study by Kamerkar et al. [73] sought to fill this important gap by comparing two siRNA delivery platforms: exosomes versus liposomes. Exosomes obtained from the supernatant of normal fibroblast-like mesenchymal cells, carrying siRNA or shRNA directed against mutated oncogenic KRASG12D (iExosomes), exhibited superior evasion of phagocytic clearance by monocytes in the circulation together with enhanced efficacy compared with similar assembled liposomes. This iExosomes treatment, mediated by a CD47-dependent phagocytotic escape mechanism, significantly extended the overall survival of treated mice in a rodent model of pancreatic cancer.

The possibility for siRNAs to achieve clinical relevance in cancer therapy suggested by this study emphasizes the key role of proteins naturally present on the surface of heterologous non-cancer cells exosomes, not only in escaping clearing mechanisms, but especially in enhancing the target specificity of exosomes as delivery systems for siRNAs directed against undruggable targets.

Several clinical trials testing siRNA-based drugs against metastatic cancers are currently underway or have been completed. A search on the portal https://clinicaltrials.gov accessed on 28 May 2025, querying with the entry “metastatic cancer and siRNA”, retrieved seven clinical studies in US (Table 1). Of these, five have been completed, one has been discontinued, and one is currently ongoing. The ongoing phase 1 study (NCT03608631) is investigating the safety and preliminary efficacy of mesenchymal stromal cell-derived iExosomes (iEXs) loaded with KRASG12D siRNA in patients with metastatic pancreatic ductal adenocarcinoma harboring the KRASG12D mutation. The results are expected in 2027. Moreover, a completed phase 1 study (NCT00672542) in metastatic melanoma tested the safety of transfected modified siRNA, decorated with antigens derived from dendritic cells, targeting the inducible immunoproteasome subunits LMP2, LMP7, and MECL1. Another phase 1 trial, named APN401 (NCT02166255), investigated the side effects and best dose of siRNA-transfected autologous peripheral blood mononuclear cells (PBMCs) in treating patients with metastatic melanoma, kidney, and pancreatic cancer. A more recent phase 1 clinical study (NCT03087591) focused on evaluating the safety and immunological activity of multiple APN401 infusions in patients with advanced solid tumors, e.g., late-stage pancreatic cancer, and its results are available. The treatment was found to induce significant immunological activation without increasing the risk of autoimmunity or systemic toxicity. Moreover, a first phase 1/2 study assessed the safety, tolerability, and toxicity of a single intravitreal injection of siRNA-027 (AGN211745) (NCT00363714), a siRNA targeting VEGFR-1, together with associated anatomical changes in the retina and in visual acuity in age-related macular degeneration (AMD). The purpose of another phase 1 trial (NCT01437007) was to test the safety and effectiveness of TKM-080301, a PLK1 siRNA, for treating primary or secondary liver cancer that has not responded to standard treatments. The last phase 1 trial (NCT02110563) aimed to test the safety and tolerability of the new drug DCR-MYC, a novel synthetic double-stranded RNA formulated as a lipid particle suspension, targeting the oncogene MYC, whose activation regulates a wide array of cellular events deeply involved in many hematologic and solid metastatic tumors. Except for one, none of the completed clinical trials have posted results on the ClinicalTrials.gov database.

Despite these limited and poorly informative clinical trials, we believe that the impact of siRNA-based drugs will play an increasingly pivotal role in the pharmacological treatment of metastatic cancers in the near future. The recent improvements and refinements of new formulations [74], together with the development of a sequence-specific control of gene expression on a genomic wide scale, termed CRISPR interference (CRISPRi) [75], will provide a very selective and complementary approach to siRNA in the targeted silencing of metastatic genes. Finally, increased knowledge of the key parameters involved in siRNA design, gained from the commercialization of several siRNA-based drugs [76] and from the technology used for the SARS-COVID-19 mRNA vaccines encapsulated in LNP, will likely boost the development and approval of siRNA-based anticancer drugs.

**Table 1:** Summary of clinical trials found on ClinicalTrials.gov, querying with the entry “metastatic cancer and siRNA”. iP: immunoproteosome; TAA: tumor-associated antigen.

Clinical Trials NCT Number	Status and Study Start	Drug or Biological	Biological Function	Diseases	Results	References
NCT03608631	Active, not recruiting, 27 January 2021	Drug: Mesenchymal stromal cells (MSC)-derived exosomes (iEXs) loaded with KRASG12D siRNA	siRNA silences KRASG12D oncogene mutation to inhibit cancer cell proliferation and survival.	Metastatic pancreatic ductal adenocarcinoma	Results are expected in 2027.	[73,77]
NCT00672542	Completed, January 2008	Biological: vaccine of autologous dendritic cells (DCs) transfected with iP-targeting siRNAs and TAA-encoding RNAs	siRNA targeting iP beta subunits (LMP2, LMP7, and MECL1) is used to modify the expression of iP-mediated antigen processed by dendritic cells, in combination with RNAs encoding melanoma TAAs (MART-1, tyrosinase, gp100, and MAGE-3), to enhance antigen-specific T cell responses.	Metastatic melanoma	The open-access abstract available in PubMed summarizes the results, mentioning two treated subjects as partially and completely responsive. No results are published on ClinicalTrials.gov	[77,78]
NCT02166255	Completed, December 2014	Biological: autologous peripheral blood mononuclear cells (PBMCs) transfected with CBLB siRNA (APN401)	siRNA silences CBLB mRNA to enhance T cells function by reducing CBLB brake. CBLB is an E3 ubiquitin ligase that functions as an intracellular checkpoint restraining lymphocyte activation.	Advanced solid tumors, e.g., metastatic melanoma, metastatic kidney cancer, and metastatic pancreatic cancer	The meeting abstract available in BMJ Journal reported that APN401 infusion seems feasible and well-tolerated. No results are published on ClinicalTrials.gov	[77,79]
NCT03087591	Completed, 28 April 2017	Biological: autologous peripheral blood mononuclear cells (PBMCs) transfected with CBLB siRNA (APN401)	siRNA silences CBLB mRNA to enhance T cells function by reducing CBLB brake. CBLB is an E3 ubiquitin ligase that functions as an intracellular checkpoint restraining lymphocyte activation.	Advanced solid tumors, e.g., metastatic pancreatic and metastatic colorectal cancer	The treatment was found to induce significant immunological activation without increasing the risk of autoimmunity or systemic toxicity. No deaths were reported; 82% of participants completed the trial, one subject was removed, and another withdrew.	[77]
NCT00363714	Completed, November 2004	Drug: siRNA-027 (AGN211745)	siRNA-027 targets and silences VEGFR1 mRNA, thereby inhibiting macular neovascularization development.	Age-related macular degeneration (AMD)	The article in AJO reported that siRNA-027 was well-tolerated with no dose-limiting effects, while visual acuity and foveal thickness were stabilized or improved. No results are published on ClinicalTrials.gov	[77,80]
NCT01437007	Completed, 26 August 2011	Drug: Lipid nanoparticle (LNP) formulated with PLK1 siRNA (TKM-080301)	siRNA targeting the polo-like kinase-1 (PLK1) gene impairs cancer cell proliferation, leading to mitotic arrest and apoptosis.	Primary or secondary liver cancer, e.g., colorectal liver metastasis, pancreas liver metastasis	The trial was limited to one participant, and, unfortunately, no results were published.	[69,77]
NCT02110563	Terminated, April 2014	Drug: Lipid nanoparticle (LNP) formulated with MYC siRNA (DCR-MYC)	siRNA silences the mRNA expression of the oncogene c-MYC, thereby inhibiting cancer cell proliferation, differentiation, and apoptosis.	Solid tumors, multiple myeloma, and lymphoma	Failed to meet therapeutic criteria	[77,81,82]

## 4. Conclusions and Future Perspectives

Over the last decade, the development of numerous anticancer drugs has markedly improved survival in patients with various solid tumors and hematologic malignancies. Yet, this progress has not been matched by effective therapies for metastatic cancer, which still accounts for over 90% of cancer-related deaths. This gap largely reflects our limited understanding of the molecular mechanisms driving metastasis, complicating the identification of actionable targets [83].

RNA-based therapeutic strategies, particularly those involving small interfering RNAs (siRNAs) and microRNAs (miRNAs), are emerging as promising tools to selectively silence key metastatic genes. Delivered directly or via advanced platforms such as lipid nanoparticles and exosomes, these molecules offer new avenues to tackle metastatic disease. While significant challenges remain, siRNA- and miRNA-based approaches have the potential to become cornerstone therapies in the fight against cancer.

Advancing the study of metastasis will require continued collaborative efforts across the oncology community to develop novel approaches and refine existing technologies, ultimately translating molecular insights into more effective treatments.

In our opinion, we foresee that the greatest improvements will derive from the combination of two complementary and intertwined approaches; (a) new types of functional ex vivo metastatic models and (b) single-cell spatial multiomic analyses.

Ombrato and colleagues [84] proposed an elegant approach to detect and identify early events in cancer cell metastasis, as well as the ability of tumor cells to recruit and “educate” nearby non-cancer cells in the premetastatic niche. In this study, premetastatic cancer cells were engineered to express a releasable mCherry fluorescent protein, which is subsequently taken up by non-tumor cells in a tissue slice representing the local tumor microenvironment (TME). Positive mCherry TME cells can be first isolated by fluorescence-activated cell sorting (FACS) and then characterized by single-cell transcriptomics and proteomics tools to identify changes in signaling pathways induced by the direct contact with metastatic cancer cells. Following this strategy, Massara et al. [85] applied the mCherry fluorescent label to a mouse model of brain metastasis and found that CD206+ tumor-associated macrophages were the predominant cell type capturing and retaining the tumor-derived marker, both adjacent to and distant from the metastatic lesion.

The full exploitation of this ex vivo model requires the implementation of reliable spatial single cell multiomic analyses, thus allowing for characterization at the genomic, transcriptomic, and proteomic levels of the different clusters of cells, along with their spatial localization, belonging to the metastatic niche and TME [86]. Recent improvements in analytical procedures have clearly shown that single-cell sequencing (SCS) is a powerful and affordable high-throughput tool to investigate the molecular mechanisms underlying tumor metastasis at the single-cell level [87]. Indeed, SCS can be used to study tumor heterogeneity, drug resistance, changes in the TME, analysis of circulating tumor cells (CTCs) in liquid biopsy, and, in combination with artificial intelligence (AI), to construct metastasis-related cell maps for predicting and monitoring the dynamics of metastasis.
